# Functional Traits Are Good Predictors of Tree Species Abundance Across 101 Subtropical Forest Species in China

**DOI:** 10.3389/fpls.2021.541577

**Published:** 2021-06-30

**Authors:** Ronghua Li, Shidan Zhu, Juyu Lian, Hui Zhang, Hui Liu, Wanhui Ye, Qing Ye

**Affiliations:** ^1^College of Natural Resources and Environment, South China Agricultural University, Guangzhou, China; ^2^Key Laboratory of Vegetation Restoration and Management of Degraded Ecosystems, South China Botanical Garden, Chinese Academy of Sciences, Guangzhou, China; ^3^Guangdong Provincial Key Laboratory of Applied Botany, South China Botanical Garden, Chinese Academy of Sciences, Guangzhou, China; ^4^Guangxi Key Laboratory of Forest Ecology and Conservation, College of Forestry, Guangxi University, Nanning, China; ^5^Institute of Tropical Agriculture and Forestry, Hainan University, Haikou, China

**Keywords:** functional traits, species abundance, leaf nutrient content, specific leaf area, wood density, hydraulic conductivity, photosynthesis, drought tolerant

## Abstract

What causes variation in species abundance for a given site remains a central question in community ecology. Foundational to trait-based ecology is the expectation that functional traits determine species abundance. However, the relative success of using functional traits to predict relative abundance is questionable. One reason is that the diversity in plant function is greater than that characterized by the few most commonly and easily measurable traits. Here, we measured 10 functional traits and the stem density of 101 woody plant species in a 200,000 m^2^ permanent, mature, subtropical forest plot (high precipitation and high nitrogen, but generally light- and phosphorus-limited) in southern China to determine how well relative species abundance could be predicted by functional traits. We found that: (1) leaf phosphorus content, specific leaf area, maximum CO_2_ assimilation rate, maximum stomata conductance, and stem hydraulic conductivity were significantly and negatively associated with species abundance, (2) the ratio of leaf nitrogen content to leaf phosphorus content (*N:P*) and wood density were significantly positively correlated with species abundance; (3) neither leaf nitrogen content nor leaf turgor loss point were related to species abundance; (4) a combination of *N:P* and maximum stomata conductance accounted for 44% of the variation in species’ abundances. Taken together, our findings suggested that the combination of these functional traits are powerful predictors of species abundance. Species with a resource-conservative strategy that invest more in their tissues are dominant in the mature, subtropical, evergreen forest.

## Introduction

Understanding the determinants of species’ relative abundances in a given habitat has long been a central question in community ecology (Preston, 1948; Silvertown, 2004; Cornwell and Ackerly, 2010; Umaña et al., 2015; Wang et al., 2021). One common approach to studying co-occurring species has involved characterizing performance-related functional traits, which can be defined as morphological, physiological, or phenological traits that influence species’ adaptations to their biotic and abiotic environments (Silvertown, 2004; McGill et al., 2007; Violle et al., 2007; Garnier and Navas, 2012; Paradis and Schliep, 2019). Functional traits have been shown to play a crucial role in the success or failure of species under specific environmental conditions (Kraft et al., 2008; Valladares et al., 2015).

Ecologists have used functional traits to detect performance differences among species, and to assess the extent to which these differences have adaptive value, thus resulting in differences in relative abundances of co-existing species (Silvertown, 2004; McGill et al., 2007; Poorter et al., 2008; Cornwell and Ackerly, 2010; Garnier and Navas, 2012). For example, studies in a water- and nitrogen-limited grassland in Greece found that low specific leaf area, leaf water content, mineral content, photosynthetic carbon assimilation, and high water use efficiency led to higher species abundances (Tsialtas et al., 2001, 2004). Positive correlations between species abundance and both biomass and nitrogen content were observed in grasslands in the United States (Fargione and Tilman, 2006; Stanley Harpole and Tilman, 2006). In a forest in eastern China, wood density was found to be positively correlated with species abundance, while leaf nitrogen content was negatively correlated with species abundance (Yan et al., 2013).

While many studies have used functional traits to predict species’ relative abundances in plant communities, empirical attempts have largely found little to no relationship (Murray et al., 2002; Shipley et al., 2006; Yan et al., 2013). In these studies, most of the correlations between the variance in individual traits and species’ relative abundances are relatively weak. Yang et al. (2018) argued that traits influence species performance depending on both the abiotic (climate, resource availability) and biotic (pathogen, competition) environments. For example, trait values associated with pathogen resistance will confer differing levels of demographic success depending on the occurrence of pathogens. Similarly, the relationship between drought tolerance traits and species’ abundances are expected to differ among climate regions differing in aridity or water deficit. Additionally, most studies have distilled functional diversity down to just a few core traits that are relatively easily to measure but may not directly determine the abundance of coexisting plant species. Here, we sought to quantify the nature and strength of the relationship between functional traits and species abundance in a mature subtropical, evergreen forest. We measured physiological traits, such as photosynthetic carbon assimilation capacity, stem hydraulic conductivity, and drought tolerance, all of which have direct effects on species performance and fitness (Pineda-Garcia et al., 2013; Li et al., 2015; Skelton et al., 2015; Zhu et al., 2018).

This study was conducted in an old growth, speciose, subtropical, evergreen forest in southern China. Large variation in species’ abundances, ranging from 1 to 5,996, was observed in a permanent 200,000 m^2^ plot (Lin et al., 2013). We used an extensive dataset to explore whether the variation in species abundance was related to differences in plant physiological traits. Specifically, we used a dataset of 101 tree species, which account for 96.56% of the individuals in this subtropical forest (see Supplementary Data Sheet 1), and quantified a number of key functional traits that directly or indirectly reflect different resource acquisition strategies and drought tolerance to determine to what extent functional traits can predict species’ abundances. Previous studies have found that soil phosphorus content and light are the factors most affecting plant growth and survival, while precipitation and soil nitrogen content in this study area are relatively high (Vitousek et al., 2010; Zhou et al., 2011; Lin et al., 2013). We therefore expected that fast-growing pioneer species with acquisitive traits (high leaf nutrient content, high specific leaf area, high carbon assimilation rate, high hydraulic conductivity and low wood density), which require nutrient-rich soils and sufficient light for recruitment, may be less common than slow-growing, shade-tolerant species. Additionally, due to relatively high precipitation in this area, we expected the associations between drought tolerance traits and species abundance would be weak.

## Materials and Methods

### Study Site and Plant Community Survey

The study site was located in the Dinghushan Biosphere Reserve (23°09′21′′N–23°11′30′′N, 112°32′39′′E–112°35′41′′E), about 84 km west of the city of Guangzhou, in Guangdong province of southern China. The region has a typical subtropical monsoon climate, with an average annual precipitation of 1678 mm. About 80% of the annual precipitation falls during the wet season, which lasts from April to September, resulting in a distinct seasonality (Zhou et al., 2011). A permanent 200,000 m^2^ (400 × 500 m) plot was established in the reserve in 2005. Vegetation in the plot is characterized by an old-growth (>400 years), monsoon, evergreen broadleaved forest. All living stems with a diameter at breast height (DBH) ≥ 1 cm were labeled. A total of 71 617 stems belonging to 210 species, 119 genera, and 56 families were recorded in the plot (Lin et al., 2013). Discrete and continuous abundance measures are commonly used to estimate plant abundance, such as stem density, basal area, and biomass (Anderson et al., 2012). In the present study, we calculated species’ abundances based on the total number of individuals and total basal area for each species in the plot. Species’ abundances measured by basal area are shown in Supplementary Figures 1–5 and Supplementary Table 1. We focused our measurements and analyses on 101 species, which accounted for 96.56% of all individuals in the community.

### Plant Functional Traits Measurement

We selected 10 functional traits to represent the major axes of plant functional variation (Reich, 2014). The 10 traits, when considered together, document the trade-off between fast-growing species and slow-growing species. For each species, three to five individuals with DBH comparable to the mean DBH of that species in the plot were sampled. Traits of 48 out of the 101 species have been reported previously (Zhu et al., 2013; Li et al., 2015), and traits for the other 53 species were measured in this study.

We quantified the carbon, phosphorus, and nitrogen economy of leaves by measuring specific leaf area (SLA), leaf phosphorus content per unit mass (*P*_*mass*_), leaf nitrogen content per unit mass (*N*_*mass*_) and *N*_*mass*_: *P*_*mass*_ ratio (*N:P*). These traits are considered part of the leaf economics spectrum (Reich et al., 1997; Wright et al., 2004). For leaf area measurements, 20 fully expanded, sun-exposed leaves in the canopy of three to five individuals per species were measured with a leaf area meter (Li-3000A; Li-Cor, Lincoln, NE, United States), with petioles and/or rachis removed. For shade-tolerant species growing in the understory, leaves were collected from the top of the canopy. Leaves were oven-dried at 70°C for 48 h after which dry mass was determined. SLA (cm^2^ g^–1^) was calculated as leaf area per dry mass. The oven-dried leaves were then ground to a fine powder. *N*_*mass*_ (g g^–1^) was determined by Kjeldahl analysis, and *P*_*mass*_ (g g^–1^) was measured using atomic absorption spectrophotometry.

We assessed light capture strategies by measuring maximum CO_2_ assimilation rate per unit mass (*A*_*mass*_; nmol g^–1^ s^–1^) and stomatal conductance per unit mass (*g*_*s*_; mmol g^–1^ s^–1^). Maximum CO_2_ assimilation rate per unit area (*A*_*area*_; μmol m^–2^ s^–1^) and stomatal conductance per unit area (*g*_*sa*_; mol m^–2^ s^–1^) were measured between 9:00 and 11:00 am on sunny days with a Li-6400 portable photosynthesis system (Li-6400, Li-Cor, Lincoln, NE, United States). Based on preliminary trials, the photosynthetic photon flux density was set to 1,500 μmol m^–2^ s^–1^ to ensure that leaves of all species were light-saturated. Ambient CO_2_ and air temperature were maintained at 390 μmol mol^–1^ and 28 °C, respectively. Prior to the data being recorded, leaves were exposed to these conditions for 5–10 min to allow for gas exchange flues to stabilize. For each species, five to ten fully expanded sun-exposed leaves were measured. *A*_*mass*_ was calculated as SLA × *A*_*area*_/10, and *g*_*s*_ was calculated as SLA × *g*_*sa*_/10.

We assessed biomass allocation and hydraulic capacity by measuring sapwood density and hydraulic conductivity. In brief, 5–10 healthy and leaf-bearing branches (6–8 mm in diameter, 40–60 cm long) from three to five individuals per species were cut in the early morning, sealed in black plastic bags with moist towels, and immediately transported to the laboratory. Prior to measurements, branch samples were re-cut under water and the cut ends retrimmed with a razor blade. To remove air embolisms, branch segments were perfused with a filtered (Ø 0.2 μm) 20 mmol KCl solution at a pressure of 0.1 MPa for 20 min. Each segment was then connected to a hydraulic conductivity-measurement apparatus (Sperry et al., 1988). An elevated water reservoir supplied the same perfusion solution to the segment during conductivity measurements, with a head pressure of ∼ 6 kPa. Water flow through the segment was allowed to equilibrate for ∼10 min, after which the mass of water flux through the segment over time (in seconds) was measured. The maximum hydraulic conductivity of the segment (*K*_*h*_) was calculated as *K*_*h*_ = *FL*/Δ*P*, where *F* is the flow rate (kg s^–1^), Δ*P* is the pressure gradient (MPa) through the segment, and *L* is the length of the segment (m). Sapwood area was quantified as the stem diameter (without bark) minus pith diameter. Sapwood-specific conductivity (*K*_*S*_; kg m^–1^ s^–1^ MPa^–1^) is equivalent to *K*_*h*_ divided by the mean of the sapwood cross-sectional areas of the two ends of the branch segment. Leaf area of all the leaves distal to the branch segment was measured with a leaf area meter (Li-3000A; Li-Cor, Lincoln, NE, United States). Leaf-specific hydraulic conductivity (*K*_*L*_; kg m^–1^ s^–1^ MPa^–1^) was calculated as the ratio of *K*_*h*_ to the leaf area distal to the branch. The sapwood density (WD; g cm^–3^) was determined from the same branch segments as used for the hydraulic conductivity measurements. The volume of fresh sapwood (with bark and pith removed) was determined by the water displacement method (Poorter et al., 2010), and the dry mass of the sapwood chunk was determined after oven-drying at 70°C for 72 h. WD was calculated as the ratio of dry mass to fresh volume.

We quantified leaf drought tolerance by measuring leaf turgor loss point (ψ_*tlp*_). For leaf pressure-volume relationships, leaf-bearing branches from three to five individuals of each species were harvested in the early morning, when leaf water potentials are high. These branches were transferred to the laboratory, where the basal ends of the branches were recut and immersed in distilled water and allowed to rehydrate while the leafy shoots were covered with plastic. For pressure-volume curve measurements, we used terminal leaves that had not been in contact with water. Leaves were first weighed to obtain the initial fresh mass and then immediately placed in a pressure chamber to determine the initial water potential. Leaf mass and water potential were measured periodically while leaves slowly desiccated in the laboratory. Leaves were eventually oven-dried for 48 h at 70°C to determine their dry mass. The leaf water potential at the turgor loss point (ψ_*tlp*_) was determined with a pressure-volume relationship analysis program developed by Schulte and Hinckley (1985).

### Statistical Analysis

All data were analyzed in R 4.0.3 (R Core Team, 2015) and are provided in the Supplementary Data Sheet 1. Prior to analyses, functional traits and species’ abundances were first log-transformed to normalize the data, which reduced the influence of species with extreme values. If the variable had negative values such as leaf turgor loss point, absolute values were used. To account for shared evolutionary history and its effects on species traits, we performed phylogenetically informed statistical tests. We used a dated phylogeny for seed plants (Smith and Brown, 2018). The phylogenetic tree was built using the phylo.maker function in the R package V.PhyloMaker (Jin and Qian, 2019). To test whether species’ abundances could be predicted by individual functional traits, we performed simple linear regressions based on phylogenetic independent contrasts (PICs) (Felsenstein, 1985). To examine multivariate associations among the 10 traits across species, we performed phylogenetic principle component analysis (PPCA) using the PICs of functional traits. The relationships between species loadings on the first two PCA axes and the abundance of individual species were then analyzed with least-squares regressions. PICs were calculated using the pic function in the R package ape (Paradis and Schliep, 2019). PPCA was carried out using the PCA function in the R package FactoMineR (Lê et al., 2008).

Multiple linear regression models were used to examine the relationships between species abundance and all of the functional traits. We used the BIC statistic to select the best model (combination of predictors and their interactions). Only models including non-correlated predictors (with a variance inflation factor <3) were considered.

## Results

**FIGURE 1:**
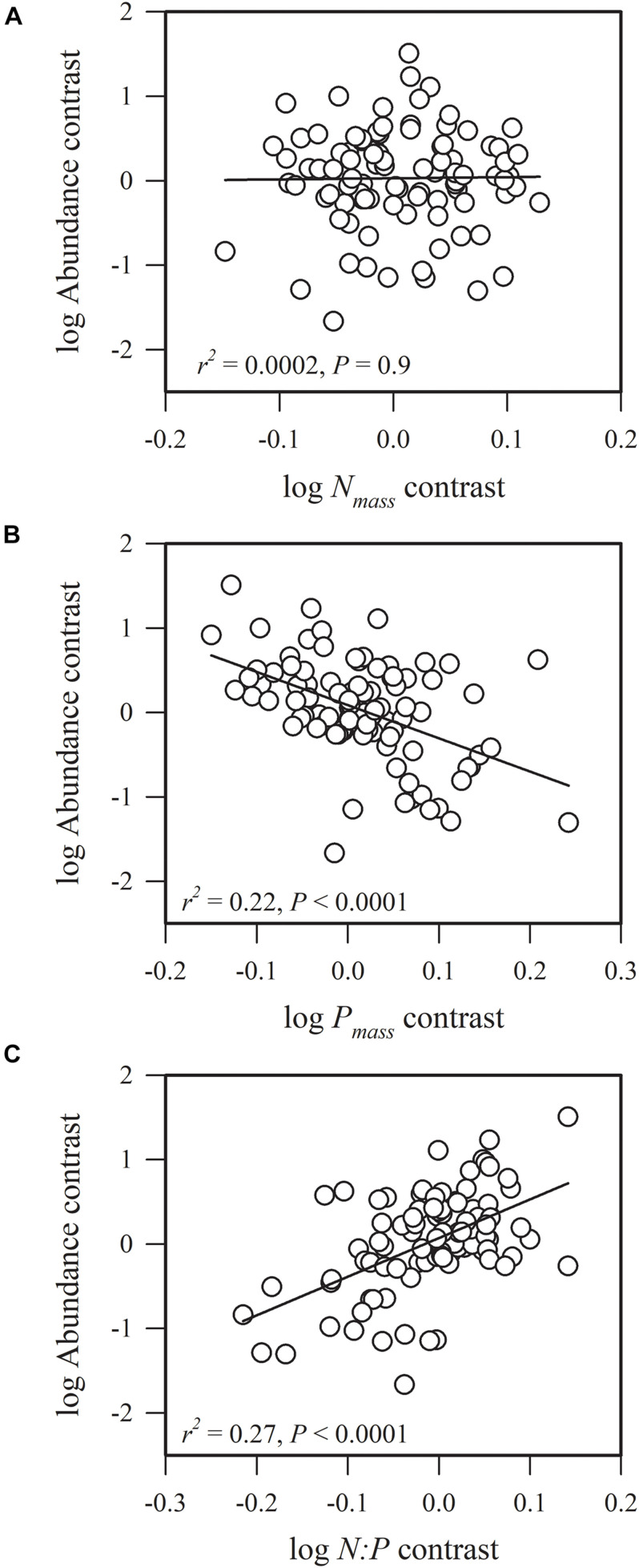
Phylogenetic correlations between species abundance and **(A)** leaf nitrogen concentration (*N*_*mass*_), **(B)** leaf phosphorus concentration (*P*_*mass*_), and **(C)** leaf nitrogen: leaf phosphorous content (*N:P*).

**FIGURE 2:**
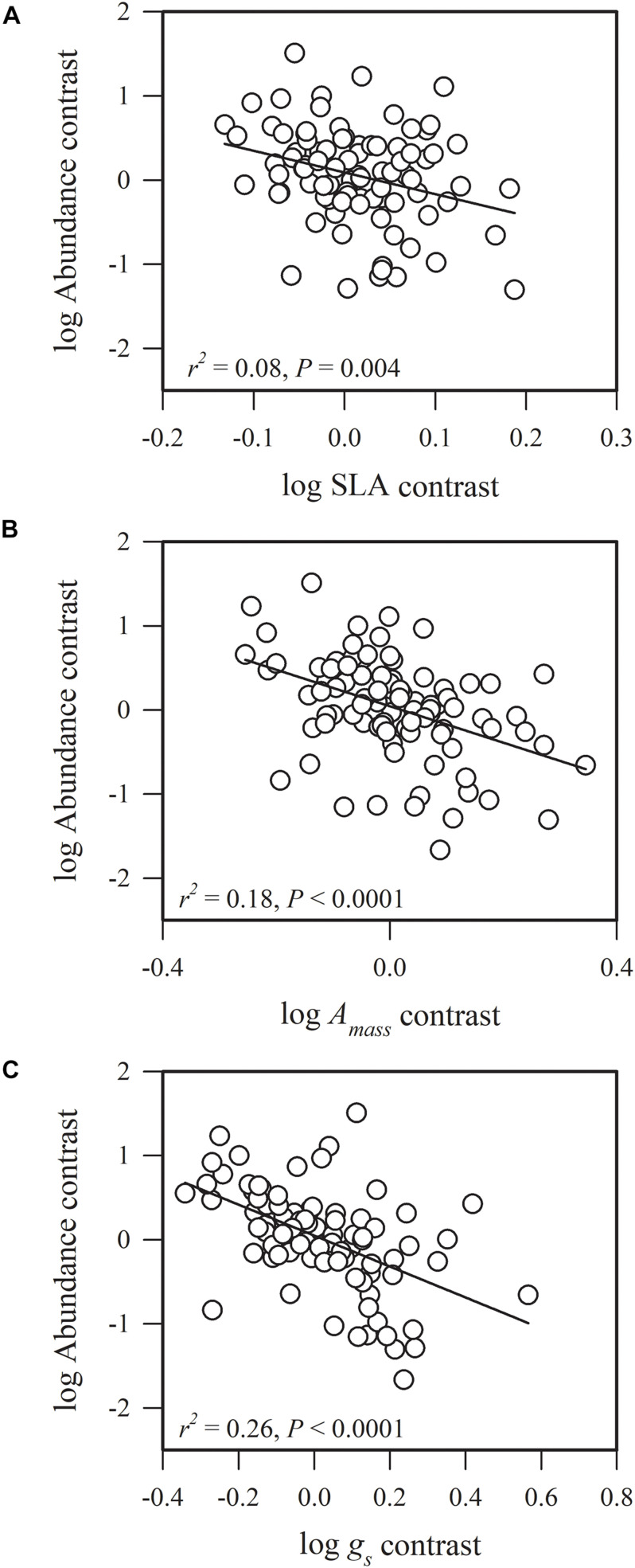
Phylogenetic correlations between species abundance and **(A)** specific leaf area (SLA), **(B)** maximum CO_2_ assimilation rate per unit mass (*A*_*mass*_), and **(C)** stomatal conductance per unit mass (*g*_*s*_).

**FIGURE 3:**
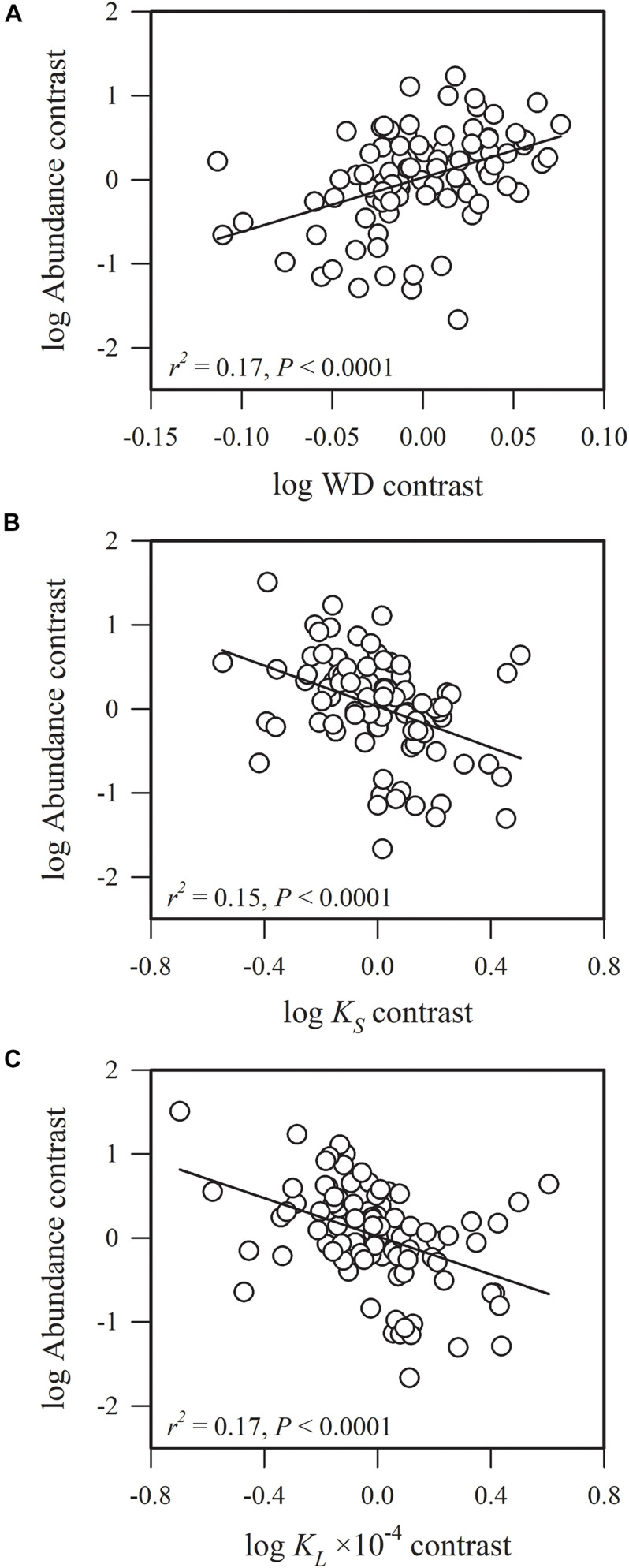
Phylogenetic correlations between species abundance and **(A)** sapwood density (WD), **(B)** sapwood-specific hydraulic conductivity (*K*_*S*_) and **(C)** leaf-specific hydraulic conductivity (*K*_*L*_).

**FIGURE 4:**
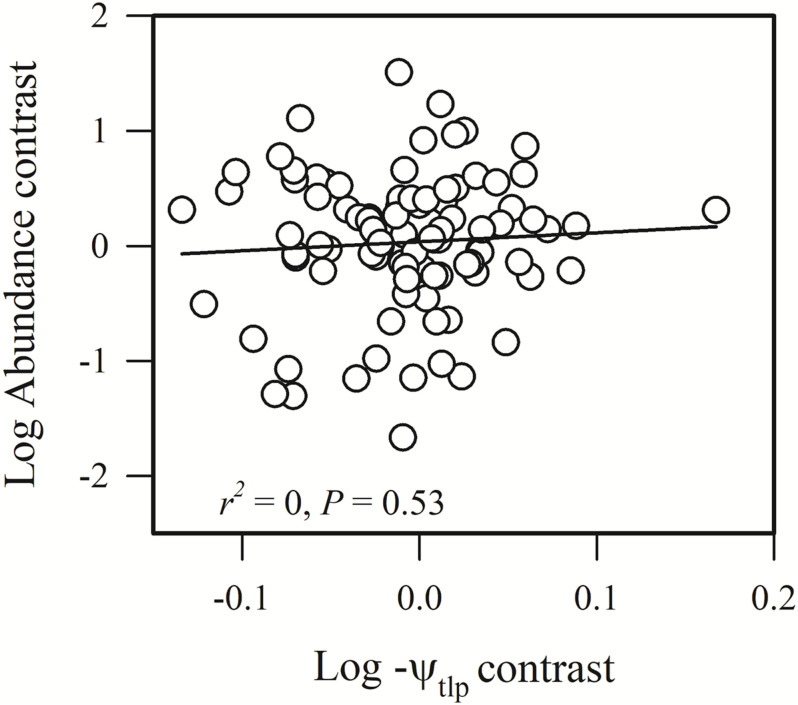
Phylogenetic correlation between species abundance and turgor loss point (–ψ_*tlp*_).

**FIGURE 5:**
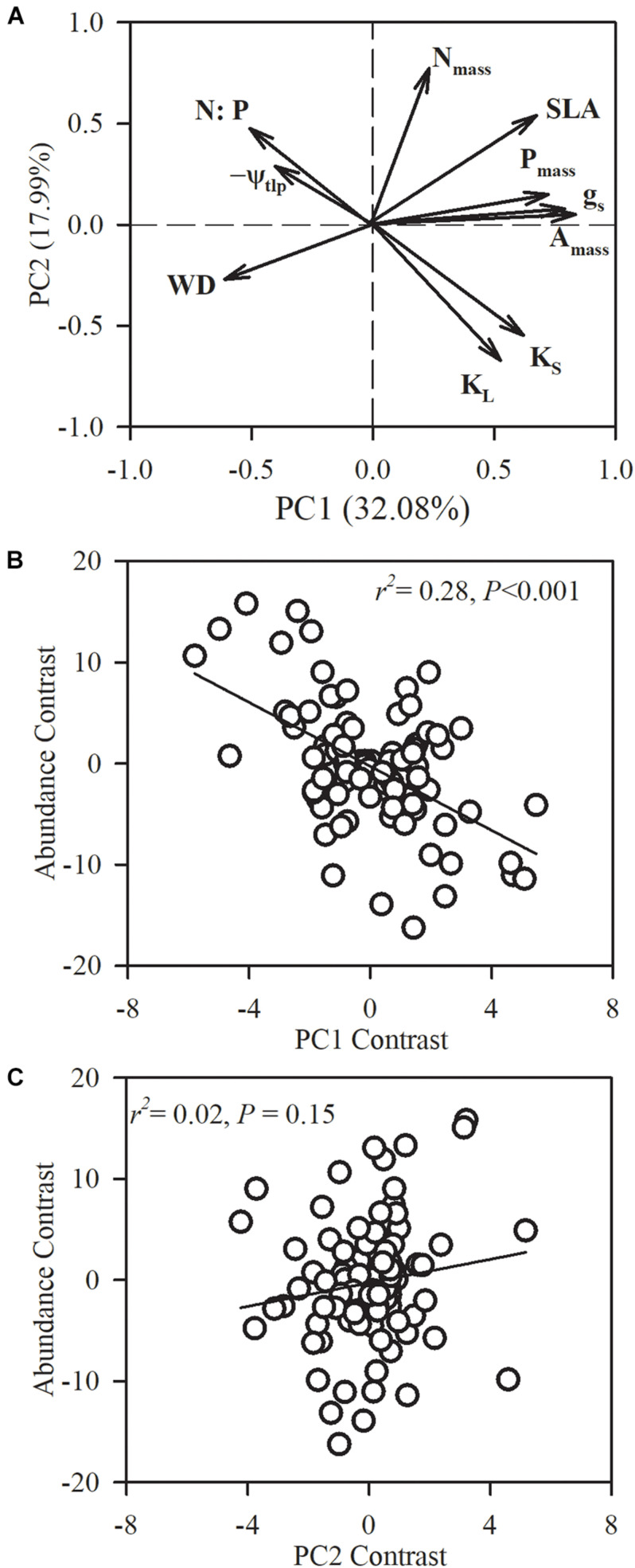
Phylogenetic principal component analysis (PPCA) for the first two principal components (PC) on the 10 functional traits of the 101 studied species. **(A)** Loading plots for the first two axes and species loadings on the first and second axes. **(B,C)** Relationships between species loadings on the first two axes and species abundance. *N*_*mass*_, leaf nitrogen concentration; *P*_*mass*_, leaf phosphorus concentration; leaf phosphorous content (*N:P*); SLA, specific leaf area; *A*_*mass*_, maximum CO_2_ assimilation rate per unit mass; *g*_*s*_, stomatal conductance per unit mass; WD, sapwood density; *K*_*S*_, sapwood-specific hydraulic conductivity; leaf-specific hydraulic conductivity (*K*_*L*_); ψ_*tlp*_, turgor loss point.

Most of the phylogenetic correlations between functional traits and species abundance (stem density) were statistically significant (Figures 1–5). In these relationships, traits explained between 0 and 27% of interspecific variation in abundance.

We found that species abundance was not significantly correlated with *N*_*mass*_ (*r*^2^ = 0.0002, *P* = 0.9), but was significantly and negatively associated with *P*_*mass*_ (*r*^2^ = 0.22, *P* < 0.0001) and was significantly and positively correlated with *N:P* (*r*^2^ = 0.27, *P* < 0.0001) (Figure 1). This result indicates that species with conservative phosphorus use strategies are highly abundant. The ratio of *N:P* ranged from 11.92 to 42.25, and 89.1% of all species had a *N:P* > 16, suggesting that the community was phosphorous-limited (Supplementary Data Sheet 1).

Species abundance and SLA were significantly and negatively related (*r*^2^ = 0.08, *P* = 0.0002) (Figure 2A), indicating that species with a low specific leaf area have a high abundance. Species abundance was significantly and negatively related to both *A*_*mass*_ (*r*^2^ = 0.18, *P* < 0.0001) and *g*_*s*_ (*r*^2^ = 0.26, *P* < 0.0001) (Figures 2B,C), suggesting that relatively higher carbon acquisition rates do not lead to a higher species abundance. Similar patterns were found for hydraulic traits, where *K*_*S*_ (*r*^2^ = 0.15, *P* < 0.0001) and *K*_*L*_ (*r*^2^ = 0.17, *P* < 0.0001) were significantly and negatively correlated with species abundance (Figures 3B,C). Species abundance was significantly and positively correlated with WD (*r*^2^ = 0.17, *P* < 0.0001) (Figure 3A), indicating that species with relatively high biomass allocation to their stems were more abundant. However, species abundance was not significantly correlated with −ψ_*tlp*_ (Figure 4), indicating that leaf drought tolerance did not influence species abundance.

In order to assess multivariate strategies of species, we conducted a PCA on the 10 functional traits across the 101 species. The first two principal components captured 50.07% of the variance, with 32.08% in the first axis (Figure 5). The first PCA axis had strong positive loadings for leaf nutrient content (*P*_*mass*_), stem hydraulic conductivity (*K*_*S*_ and *K*_*L*_) and photosynthetic-related traits (*g*_*s*_ and *A*_*mass*_), and negative loadings for WD, *N:P* and −ψ_*tlp*_. The second PCA axis explained 17.99% of the variation, and was primarily driven by stem hydraulic conductivity (*K*_*S*_ and *K*_*L*_) and *N*_*mass*_. We found that species abundance was significantly correlated with species loadings on the first axis (*r*^2^ = 0.28, *P* < 0.0001).

The best multiple regression model explained 44% of the variation in species abundance. The model included *g*_*s*_ and *N:P* (Table 1) and shows that functional traits account for a relatively large proportion of variation in species abundance.

**TABLE 1:** Results of the multiple regression for plant functional traits and species abundance based on PICs.

Models	*N*	*B*	SE *B*	t	*F*	*R*^2^	*P*
*g_*s*_* + *N:P*	93				36.87	0.44	<0.0001
*g*_*s*_		−1.53	0.28	−5.35			<0.0001
*N:P*		3.84	00.69	5.52			<0.0001

*Stomatal conductance per unit mass (*g*_*s*_); *N:P* (*N*_*mass*_:*P*_*mass*_).*

Analyses of species abundance measured by basal area showed similar results as stem density (Supplementary Figures 1–5 and Supplementary Table 1). Most of the relationships did not change significantly, however, the proportion of variance in species abundance explained changed. *N*_*mass*_, *K*_*S*_ and ψ_*tlp*_ were not significantly correlated with basal area, while *P*_*mass*_, SLA, *A*_*mass*_, *g*_*s*_, and *K*_*L*_ were significantly and negatively correlated with basal area. *N:P* and WD were significantly positively correlated with basal area.

## Discussion

In the present study, our results showed that individual functional traits accounted for 0–27% of the variance in species abundance, while the two functional traits included in the best model (i.e., *g*_*s*_ and *N:P*) accounted for 44% of the variance. We found that slow-growing species with a low nutrient content (low *P*_*mass*_) and high *N:P*, high resource input into construction (low SLA and high WD), low carbon fixation rates (low *A*_*mass*_ and *g*_*s*_), and low hydraulic conductivity (low *K*_*S*_ and *K*_*L*_) have more individuals than fast-growing species with the opposite trait patterns. Additionally, leaf drought tolerance did not affect species abundance. These results revealed that a relatively low carbon assimilation rate, low water transport efficiency, and a conservative nutrient use strategy contributed to high species abundance.

In phosphorus-limited subtropical forests, species that possess traits associated with adaption to phosphorous limitation would be predicted to have higher abundances. Indeed, we found species with relatively low *P*_*mass*_ and high *N:P* ratio, traits associated with adaption to phosphorous limitation, to be dominant in the forest. This result was supported by previous work, which found that plants in phosphorus-limited sites are characterized by relatively low leaf phosphorus contents and high *N:P* ratios (Lambers et al., 2010). The mean leaf *N:P* for the species we studied was 23.45, indicating strong P-limitation (Koerselman and Meuleman, 1996). Relatively low nutrient contents in tissues may reduce the demand for limited resources and provide more structural tissue for the same amount of nutrients (Chapin, 1980; Lambers et al., 2010). Additionally, low nutrient content is often associated with higher physical and chemical defenses in the tissues, which can reduce the risk of being attacked by microorganisms and herbivores (Coley and Barone, 1996). However, *N*_*mass*_ was found to not significantly correlate with species abundance, likely due to relatively high soil nitrogen content and nitrogen deposition in the study region (Fang et al., 2009).

The other two traits we found to be significant in our study (WD and SLA) are important in carbon allocation, which underlies much of the variation in other functional traits (Osnas et al., 2013; Supplementary Figure 6). High WD and low SLA lead to a relatively high carbon allocation toward structural support, a low growth rate, a high resistance to mechanical damage, and a high resistance to pathogenic and fungal attack, all of which decrease the risk of damage and death to plants (Clark and Clark, 2001; Poorter et al., 2010; Falster et al., 2011). Recently, Paradis and Schliep (2019) found that species with high WD are able to tolerate competition and are more competitive than their neighbors, while species with low SLA are highly competitive with their neighbors. Thus, species with a low WD and high SLA grow faster and may have advantages in the short-term, while species with well-defended, high-density stems and a low SLA may have advantages over the long-term (Rüger et al., 2012).

Many studies have shown that plant physiological traits, such as plant carbon acquisition and hydraulic conductivity, are crucial for species survival and growth (Kraft and Ackerly, 2010; Poorter et al., 2010; McDowell, 2011; Adler et al., 2014). Relatively few studies, however, have tested whether these traits can affect species’ abundances in a forest community. We tested whether physiological traits (e.g., carbon acquisition, hydraulic conductivity, and drought tolerance) were associated with species abundance. We found that carbon acquisition rate and hydraulic conductivity rate were significantly negatively correlated with species abundance (*P* < 0.001), while traits associated with drought tolerance were not significantly correlated with species abundance.

It is not surprising that high carbon assimilation, high stomatal conductance, and high hydraulic conductivity did not lead to high species abundance in our forest. Instead, plants that employ a conservation strategy are more successful than those focusing on a higher efficiency of carbon gain in shady environments in old-growth forests (Baltzer and Thomas, 2007; Lusk et al., 2011). Moreover, according to the resource availability hypothesis, species adapted to resource-poor environments usually grow more slowly, and invest more in constitutive defenses and construction (Endara and Coley, 2011). Notwithstanding what is limiting in a specific environment (e.g., light, nutrients, water), a fast or slow growth strategy results in a similar set of leaf and stem traits (Reich, 2014). In the old growth forest we studied, superior competitors that are able to persist in a resource-limited environment (low soil phosphorus content and low light), possess traits such as low*P_*mass*_*, low SLA, and high WD. These traits also led to a low stomatal conductance, low carbon assimilation rate, and low hydraulic conductivity (Supplementary Figure 6). However, drought tolerance did not influence variation in species abundance in the old-growth forest, likely due to the low likelihood of experiencing physiological drought because of high precipitation and high atmospheric humidity. When water is not a limiting factor, drought tolerance traits tend to be convergent and not divergent in plant communities (Garnier and Navas, 2012).

Collectively, our findings suggest that plant traits are strong predictors of species’ abundances. We confirmed the prediction that dominant species in mature forests grow more slowly and invest constitutively more in their tissues. Along a rank-abundance curve, dominance declines in association with a transition from a resource-conservative strategy to a resource-acquisitive strategy. Stable community structure is the result of a dynamic equilibrium, and in a mature forest this is reflected in patches with different successional age (Chambers et al., 2013). In a mature forest, gaps (e.g., due to tree falls) allow the establishment of fast-growing pioneer species with high nutrient concentrations in their tissues, high carbon assimilation rates, and high hydraulic conductivity. However, ecological succession in these gaps is associated with declines in soil nutrient content and light intensities. As a result, these fast-growing pioneer species will gradually be replaced by a sequence of slow-growing, shade-tolerant species (Tilman, 1990).

Overall, the observed pattern between functional traits and species’ abundances was possibly due to limited soil phosphorus content and/or limited light in the old-growth forest. We acknowledge potential uncertainties and limitations in this study. First, the best multiple regression model explained 44% of the variation in species abundance, meaning that the majority of the variation in species abundance (56%) remains unexplained. Clearly, other factors can influence species’ abundances, such as the biogeographic history of the region and stochastic processes associated with neutrality (Gilbert and Lechowicz, 2004; Cornwell and Ackerly, 2010). It should also be noted that the traits we measured represent only some of the characteristics of plant function. Other traits that we did not measure (e.g., seed mass, leaf life span, and root traits) may also influence species abundance. Lastly, we measured the functional traits of 101 species, accounting for 96.56% percent of the individuals found in the community. However, there were additional 3.44% individuals that were not studied, which could potentially lead to bias in our assessment of the relationships between traits and abundance.

## Data Availability Statement

All datasets generated for this study are included in the article/Supplementary Material.

## Author Contributions

QY, RL, and SZ designed the research and wrote the manuscript. RL and SZ performed the research. RL, HZ, HL, JL, and WY analyzed the data. All authors contributed to the article and approved the submitted version.

## Conflict of Interest

The authors declare that the research was conducted in the absence of any commercial or financial relationships that could be construed as a potential conflict of interest.

## Supplementary Material

Supplementary Figure 1 Phylogenetic correlations between species abundance (basal area) and **(A)** leaf nitrogen concentration (*N*_*mass*_), **(B)** leaf phosphorus concentration (*P*_*mass*_), and **(C)** leaf nitrogen: leaf phosphorous content (*N:P*).

Supplementary Figure 2 Phylogenetic correlations between species abundance (basal area) and **(A)** specific leaf area (SLA), **(B)** maximum CO_2_ assimilation rate per unit mass (*A*_*mass*_), and **(C)** stomatal conductance per unit mass (*g*_*s*_).

Supplementary Figure 3 Phylogenetic correlations between species abundance (basal area) and **(A)** sapwood density (WD), **(B)** sapwood-specific hydraulic conductivity (*K*_*S*_) and **(C)** leaf-specific hydraulic conductivity (*K*_*L*_).

Supplementary Figure 4 Phylogenetic correlation between species abundance (basal area) and turgor loss point (−ψ_*tlp*_).

Supplementary Figure 5 Phylogenetic principal component analysis (PPCA) for the first two principal components (PC) on the 10 functional traits of the 101 studied species. **(A)** Loading plots for the first two axes and species loadings on the first and second axes. **(B,C)** Relationships between species loadings on the first two axes and species abundance (basal area). *N*_*mass*_, leaf nitrogen concentration; *P*_*mass*_, leaf phosphorus concentration; leaf phosphorous content (*N:P*); SLA, specific leaf area; *A*_*mass*_, maximum CO_2_ assimilation rate per unit mass; *g*_*s*_, stomatal conductance per unit mass; WD, sapwood density; *K*_*S*_, sapwood-specific hydraulic conductivity; leaf-specific hydraulic conductivity (*K*_*L*_); ψ_*tlp*_, turgor loss point.

Supplementary Figure 6 Phylogenetic correlations between the studied traits for the 101 studied species.

Supplementary Table 1 Results of the multiple regression for plant functional traits and species abundance (basal area) based on PICS.

Supplementary Data Sheet 1 Species abundance and mean value of traits for the 101 studied tree species.

## References

[B1] Adler P. B. Salguero-Gómez R. Compagnoni A. Hsu J. S. Ray-Mukherjee J. Mbeau-Ache C. (2014). Functional traits explain variation in plant life history strategies. *Proc. Natl. Acad. Sci. U.S.A.* 111, 740–745. 10.1073/pnas.1315179111 24379395 PMC3896207

[B2] Anderson B. J. Chiarucci A. Williamson M. (2012). How differences in plant abundance measures produce different species-abundance distributions. *Method Ecol. Evol.* 3 783–786. 10.1111/j.2041-210x.2012.00229.x

[B3] Baltzer J. L. Thomas S. C. (2007). Determinants of whole-plant light requirements in Bornean rain forest tree saplings. *J. Ecol.* 95 1208–1221. 10.1111/j.1365-2745.2007.01286.x

[B4] Chambers J. Q. Negron-Juarez R. I. Marra D. M. Di Vittorio A. Tews J. Roberts D. (2013). The steady-state mosaic of disturbance and succession across an old-growth Central Amazon forest landscape. *Proc. Natl. Acad. Sci. U.S.A.* 110 3949–3954. 10.1073/pnas.1202894110 23359707 PMC3593828

[B5] Chapin F. S. III (1980). The mineral nutrition of wild plants. *Annu. Rev. Ecol. Syst.* 11 233–260. 10.1146/annurev.es.11.110180.001313

[B6] Clark D. A. Clark D. B. (2001). Getting to the canopy: tree height growth in a neotropical rain forest. *Ecology* 82 1460–1472. 10.1890/0012-9658(2001)082[1460:gttcth]2.0.co;2

[B7] Coley P. D. Barone J. A. (1996). Herbivory and plant defenses in tropical forests. *Annu. Rev. Ecol. Syst.* 27 305–335. 10.1146/annurev.ecolsys.27.1.305

[B8] Cornwell W. K. Ackerly D. D. (2010). A link between plant traits and abundance: evidence from coastal California woody plants. *J. Ecol.* 98 814–821. 10.1111/j.1365-2745.2010.01662.x

[B9] Endara M.-J. Coley P. D. (2011). The resource availability hypothesis revisited: a meta-analysis. *Funct. Ecol.* 25 389–398. 10.1111/j.1365-2435.2010.01803.x

[B10] Falster D. S. Brännström D. Dieckmann U. Westoby M. (2011). Influence of four major plant traits on average height, leaf-area cover, net primary productivity, and biomass density in single-species forests: a theoretical investigation. *J. Ecol.* 99 148–164. 10.1111/j.1365-2745.2010.01735.x

[B11] Fang Y. T. Zhu W. X. Gundersen P. Mo J. M. Zhou G. Y. Yoh M. (2009). Large loss of dissolved organic nitrogen from nitrogen-saturated forests in subtropical China. *Ecosystems* 12 33–45. 10.1007/s10021-008-9203-7

[B12] Fargione J. Tilman D. (2006). Plant species traits and capacity for resource reduction predict yield and abundance under competition in nitrogen-limited grassland. *Funct. Ecol.* 20 533–540. 10.1111/j.1365-2435.2006.01116.x

[B13] Felsenstein J. (1985). Phylogenies and the comparative method. *Am. Nat.* 125 1–15. 10.1086/284325

[B14] Garnier E. Navas M.-L. (2012). A trait-based approach to comparative functional plant ecology: concepts, methods and applications for agroecology. A review. *Agron. Sustain. Dev.* 32 365–399. 10.1007/s13593-011-0036-y

[B15] Gilbert B. Lechowicz M. J. (2004). Neutrality, niches, and dispersal in a temperate forest understory. *Proc. Natl. Acad. Sci. U.S.A.* 101 7651–7656. 10.1073/pnas.0400814101 15128948 PMC419661

[B16] Jin Y. Qian H. (2019). V.PhyloMaker: an R package that can generate very large phylogenies for vascular plants. *Ecography* 42 1353–1359. 10.1016/j.pld.2022.05.005 PMC9363651 35967255

[B17] Koerselman W. Meuleman A. F. M. (1996). The vegetation N:P ratio: a new tool to detect the nature of nutrient limitation. *J. Appl. Ecol.* 33 1441–1450. 10.2307/2404783

[B18] Kraft N. J. B. Ackerly D. D. (2010). Functional trait and phylogenetic tests of community assembly across spatial scales in an Amazonian forest. *Ecol. Monogr.* 80 401–422. 10.1890/09-1672.1

[B19] Kraft N. J. B. Valencia R. Ackerly D. D. (2008). Functional traits and niche-based tree community assembly in an Amazonian forest. *Science* 322 580–582. 10.1126/science.1160662 18948539

[B20] Lambers H. Brundrett M. Raven J. Hopper S. (2010). Plant mineral nutrition in ancient landscapes: high plant species diversity on infertile soils is linked to functional diversity for nutritional strategies. *Plant Soil* 334 11–31. 10.1007/s11104-010-0444-9

[B21] Lê S. Josse J. Husson F. (2008). FactoMineR: an R package for multivariate analysis. *J. Stat. Softw.* 25 1–18.

[B22] Li R. Zhu S. Chen H. Y. John R. Zhou G. Zhang D. (2015). Are functional traits a good predictor of global change impacts on tree species abundance dynamics in a subtropical forest? *Ecol. Lett.* 18 1181–1189. 10.1111/ele.12497 26311436

[B23] Lin G. Stralberg D. Gong G. Huang Z. Ye W. Wu L. (2013). Separating the effects of environment and space on tree species distribution: from population to community. *PLoS One* 8:e56171. 10.1371/journal.pone.0056171 23409151 PMC3568135

[B24] Lusk C. H. Pérez-Millaqueo M. M. Piper F. I. Saldaña A. (2011). Ontogeny, understorey light interception and simulated carbon gain of juvenile rainforest evergreens differing in shade tolerance. *Ann. Bot.* 108 419–428. 10.1093/aob/mcr166 21856637 PMC3158685

[B25] McDowell N. G. (2011). Mechanisms linking drought, hydraulics, carbon metabolism, and vegetation mortality. *Plant Physiol.* 155 1051–1059. 10.1104/pp.110.170704 21239620 PMC3046567

[B26] McGill B. J. Etienne R. S. Gray J. S. Alonso D. Anderson M. J. Benecha H. K. (2007). Species abundance distributions: moving beyond single prediction theories to integration within an ecological framework. *Ecol. Lett.* 10 995–1015. 10.1111/j.1461-0248.2007.01094.x 17845298

[B27] Murray B. R. Thrall P. H. Gill A. M. Nicotra A. B. (2002). How plant life-history and ecological traits relate to species rarity and commonness at varying spatial scales. *Austral. Ecol.* 27 291–310. 10.1046/j.1442-9993.2002.01181.x

[B28] Osnas J. L. D. Lichstein J. W. Reich P. B. Pacala S. W. (2013). Global leaf trait relationships: mass, area, and the leaf economics spectrum. *Science* 340 741–744. 10.1126/science.1231574 23539179

[B29] Paradis E. Schliep K. (2019). ape 5.0: an environment for modern phylogenetics and evolutionary analyses in R. *Bioinformatics* 35 526–528. 30016406 10.1093/bioinformatics/bty633

[B30] Pineda-Garcia F. Paz H. Meinzer F. C. (2013). Drought resistance in early and late secondary successional species from a tropical dry forest: the interplay between xylem resistance to embolism, sapwood water storage and leaf shedding. *Plant Cell Environ.* 36 405–418. 10.1111/j.1365-3040.2012.02582.x 22812458

[B31] Poorter L. Mcdonald I. Alarcon A. Fichtler E. Licona J. C. Pena-Claros M. (2010). The importance of wood traits and hydraulic conductance for the performance and life history strategies of 42 rainforest tree species. *New Phytol.* 185 481–492. 10.1111/j.1469-8137.2009.03092.x 19925555

[B32] Poorter L. Wright S. J. Paz H. Ackerly D. D. Condit R. Ibarra-Manríquez G. (2008). Are functional traits good predictors of demographic rates? Evidence from five neotropical forests. *Ecology* 89 1908–1920. 10.1890/07-0207.1 18705377

[B33] Preston F. W. (1948). The commonness, and rarity, of species. *Ecology* 29 254–283. 10.2307/1930989

[B34] R Core Team (2015). *R: A Language and Environment for Statistical Computing*. Vienna: R Foundation for Statistical Computing.

[B35] Reich P. B. (2014). The world-wide ‘fast–slow’ plant economics spectrum: a traits manifesto. *J. Ecol.* 102 275–301. 10.1111/1365-2745.12211

[B36] Reich P. B. Walters M. B. Ellsworth D. S. (1997). From tropics to tundra: global convergence in plant functioning. *Proc. Natl. Acad. Sci. U.S.A.* 94 13730–13734. 10.1073/pnas.94.25.13730 9391094 PMC28374

[B37] Rüger N. Wirth C. Wright S. J. Condit R. (2012). Functional traits explain light and size response of growth rates in tropical tree species. *Ecology* 93 2626–2636. 10.1890/12-0622.1 23431593

[B38] Schulte P. J. Hinckley T. M. (1985). A comparison of pressure-volume curve data-analysis techniques. *J. Exp. Bot.* 36 1590–1602. 10.1093/jxb/36.10.1590 12432039

[B39] Shipley B. Vile D. Garnier S. (2006). From plant traits to plant communities: a statistical mechanistic approach to biodiversity. *Science* 314 812–814. 10.1126/science.1131344 17023613

[B40] Silvertown J. (2004). Plant coexistence and the niche. *Trends Ecol. Evol.* 19 605–611. 10.1016/j.tree.2004.09.003

[B41] Skelton R. P. West A. G. Dawson T. E. (2015). Predicting plant vulnerability to drought in biodiverse regions using functional traits. *Proc. Natl. Acad. Sci. U.S.A.* 112 5744–5749. 10.1073/pnas.1503376112 25902534 PMC4426410

[B42] Smith S. A. Brown J. W. (2018). Constructing a broadly inclusive seed plant phylogeny. *Am. J. Bot.* 105 302–314. 10.1002/ajb2.1019 29746720

[B43] Sperry J. S. Donnelly J. R. Tyree M. T. (1988). A method for measuring hydraulic conductivity and embolism in xylem. *Plant Cell Environ.* 11 35–40. 10.1111/j.1365-3040.1988.tb01774.x

[B44] Stanley Harpole W. Tilman D. (2006). Non-neutral patterns of species abundance in grassland communities. *Ecol. Lett.* 9 15–23. 16958864 10.1111/j.1461-0248.2005.00836.x

[B45] Tilman D. (1990). Constraints and tradeoffs: toward a predictive theory of competition and succession. *Oikos* 58 3–15. 10.2307/3565355

[B46] Tsialtas J. T. Handley L. L. Kassioumi M. T. Veresoglou D. S. Gagianas A. A. (2001). Interspecific variation in potential water-use efficiency and its relation to plant species abundance in a water-limited grassland. *Funct. Ecol.* 15 605–614. 10.1046/j.0269-8463.2001.00555.x

[B47] Tsialtas J. T. Pritsa T. S. Veresoglou D. S. (2004). Leaf physiological traits and their importance for species success in a Mediterranean grassland. *Photosynthetica* 42 371–376. 10.1023/b:phot.0000046155.40940.0c

[B48] Umaña M. N. Zhang C. Cao M. Lin L. Swenson N. G. (2015). Commonness, rarity, and intraspecific variation in traits and performance in tropical tree seedlings. *Ecol. Lett.* 18:1329. 10.1111/ele.12527 26415689

[B49] Valladares F. Bastias C. C. Godoy O. Granda E. Escudero A. (2015). Species coexistence in a changing world. *Front. Plant Sci.* 6:866. 10.3389/fpls.2015.00866 26528323 PMC4604266

[B50] Violle C. Navas M.-L. Vile D. Kazakou E. Fortunel C. Hummel I. (2007). Let the concept of trait be functional! *Oikos* 116 882–892. 10.1111/j.0030-1299.2007.15559.x

[B51] Vitousek P. M. Porder S. Houlton B. Z. Chadwick O. A. (2010). Terrestrial phosphorus limitation: mechanisms, implications, and nitrogen–phosphorus interactions. *Ecol. Appl.* 20 5–15. 10.1890/08-0127.1 20349827

[B52] Wang W. Jiang Y. Li B. Xi N. Chen Y. He D. (2021). Species abundance is jointly determined by functional traits and negative density dependence in a subtropical forest in southern China. *J. Plant Ecol.* 14 491–503. 10.1093/jpe/rtab009

[B53] Wright I. J. Reich P. B. Westoby M. Ackerly D. D. Baruch Z. Bongers F. (2004). The worldwide leaf economics spectrum. *Nature* 428 821–827. 15103368 10.1038/nature02403

[B54] Yan E. R. Yang X. D. Chang S. X. Wang X. H. (2013). Plant trait-species abundance relationships vary with environmental properties in subtropical forests in eastern china. *PLoS One* 8:e61113. 10.1371/journal.pone.0061113 23560114 PMC3616145

[B55] Yang J. Cao M. Swenson N. G. (2018). Why functional traits do not predict tree demographic rates. *Trends Ecol. Evol.* 33 326–336. 10.1016/j.tree.2018.03.003 29605086

[B56] Zhou G. Wei X. Wu Y. Liu S. Huang Y. Yan J. (2011). Quantifying the hydrological responses to climate change in an intact forested small watershed in Southern China. *Glob. Change Biol.* 17 3736–3746. 10.1111/j.1365-2486.2011.02499.x

[B57] Zhu S. Song J. Li R. Ye Q. (2013). Plant hydraulics and photosynthesis of 34 woody species from different successional stages of subtropical forests. *Plant Cell Environ.* 36 879–891. 10.1111/pce.12024 23057774

[B58] Zhu S. D. He P. C. Li R. H. Fu S. L. Lin Y. B. Zhou L. X. (2018). Drought tolerance traits predict survival ratio of native tree species planted in a subtropical degraded hilly area in South China. *For. Ecol. Manag.* 41”’ 1–46. 10.1016/j.foreco.2017.09.016

