# Supplementary material for: Functional Traits Are Good Predictors of Tree Species Abundance Across 101 Subtropical Forest Species in China
Source: Front Plant Sci. 2021 Jun 30;12:541577. doi: 10.3389/fpls.2021.541577 (PMC8278196; doi:10.3389/fpls.2021.541577)
Supplement: Supplementary Figure 1 — Phylogenetic correlations between species abundance (basal area) and (A) leaf nitrogen concentration (Nmass), (B) leaf phosphorus concentration (Pmass), and (C) leaf nitrogen: leaf phosphorous content (N:P). [file Data_Sheet_2.doc]

**Figure legends**

**Fig. S1** Phylogenetic correlations between species abundance (basal area) and (A) leaf nitrogen concentration (*N_mass_*), (B) leaf phosphorus concentration (*P_mass_*), and (C) leaf nitrogen: leaf phosphorous content (*N:P*). Figures are plotted on logarithmic scales and models are fitted to log-transformed data.

**Fig. S2** Phylogenetic correlations between species abundance (basal area) and (A) specific leaf area (SLA), (B) maximum CO_2_ assimilation rate per unit mass (*A_mass_*), and (C) stomatal conductance per unit mass (*g_s_*). Figures are plotted on logarithmic scales and models are fitted to log-transformed data.

**Fig. S3** Phylogenetic correlations between species abundance (basal area) and (A) sapwood density (WD), (B) sapwood-specific hydraulic conductivity (*K_S_*) and (C) leaf-specific hydraulic conductivity (*K_L_*). Figures are plotted on logarithmic scales and models are fitted to log-transformed data.

**Fig. S4** Phylogenetic correlation between species abundance (basal area) and turgor loss point (-ψ_tlp_). Figures are plotted on logarithmic scales and models are fitted to log-transformed data.

**Fig. S5** Phylogenetic principal component analysis (PPCA) for the first two principal components (PC) on the 10 functional traits of the 101 studied species. (A) Loading plots for the first two axes and species loadings on the first and second axes. (B-C) relationships between species loadings on the first two axes and species abundance (basal area). *N_mass_*, leaf nitrogen concentration; *P_mass_*, leaf phosphorus concentration; leaf phosphorous content (*N:P*); SLA, specific leaf area; *A_mass_*, maximum CO_2_ assimilation rate per unit mass; *g_s_*, stomatal conductance per unit mass; WD, sapwood density; *K_S_*, sapwood-specific hydraulic conductivity; leaf-specific hydraulic conductivity (*K_L_*); ψ_tlp_, turgor loss point.

**Fig. S6** Phylogenetic correlations between the studied traits for the 101 studied species.

**
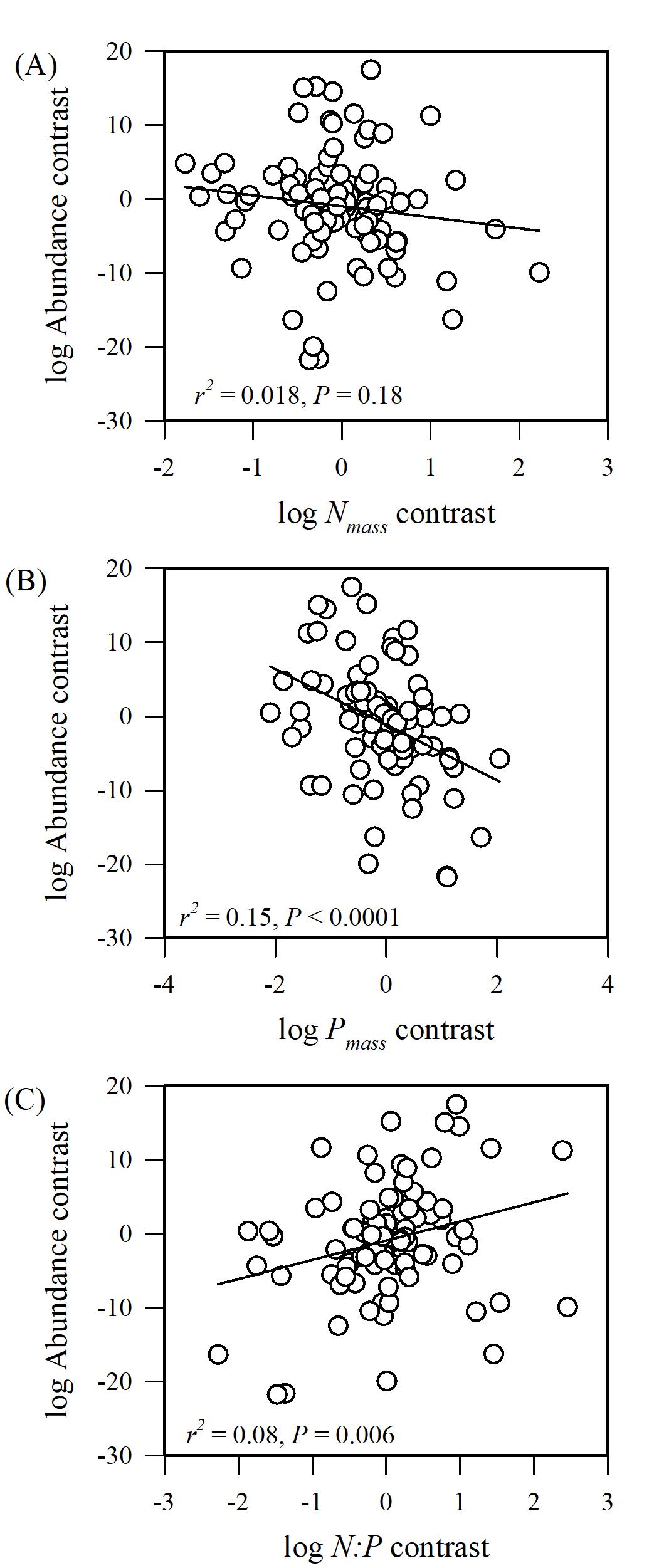
**

**Fig. S1**

**
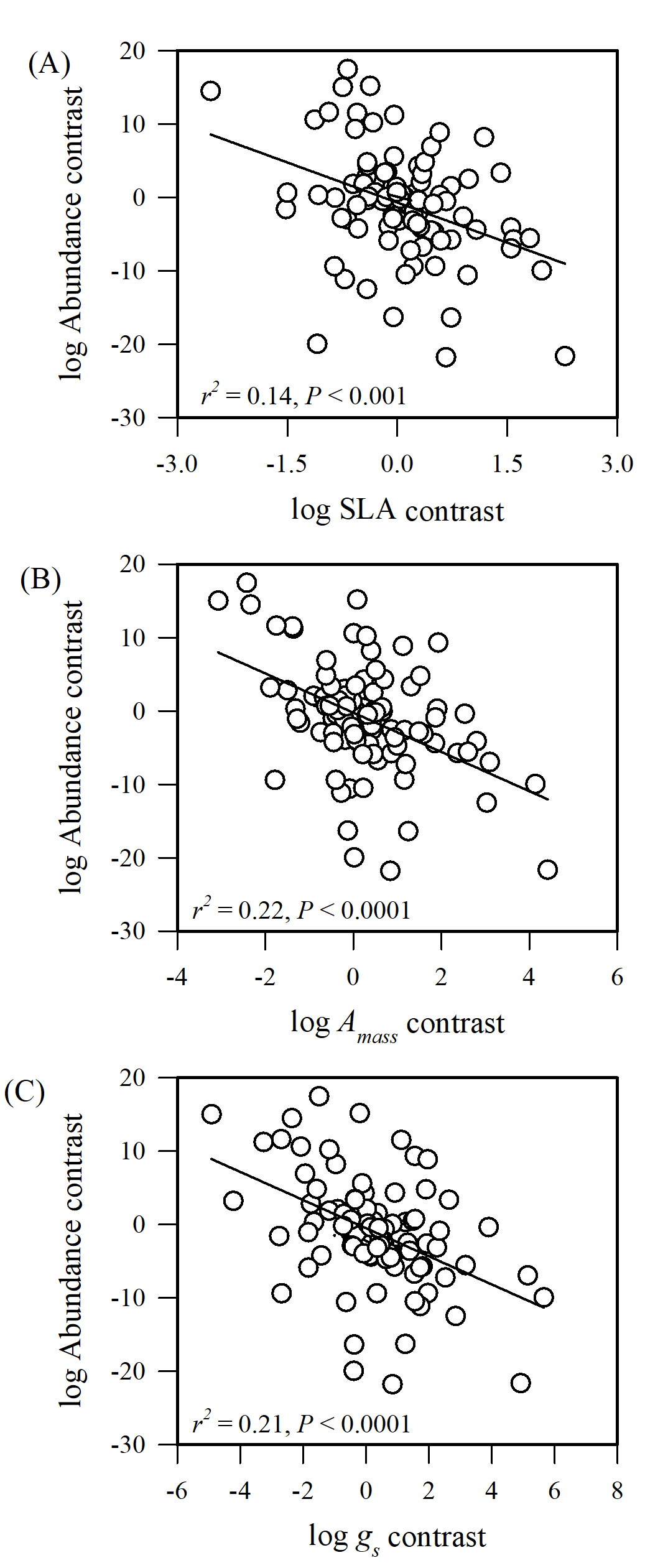
**

**Fig. S2**

**
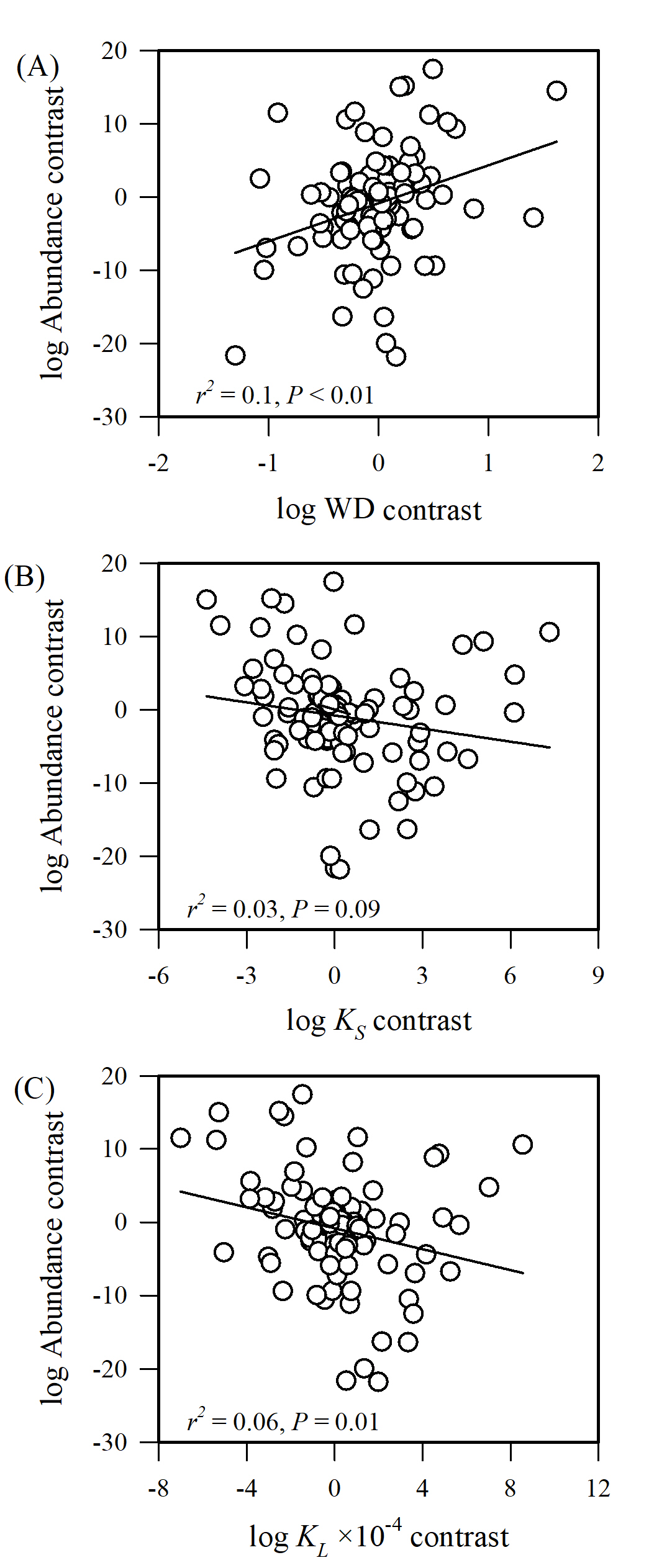
**

**Fig. S3**

**
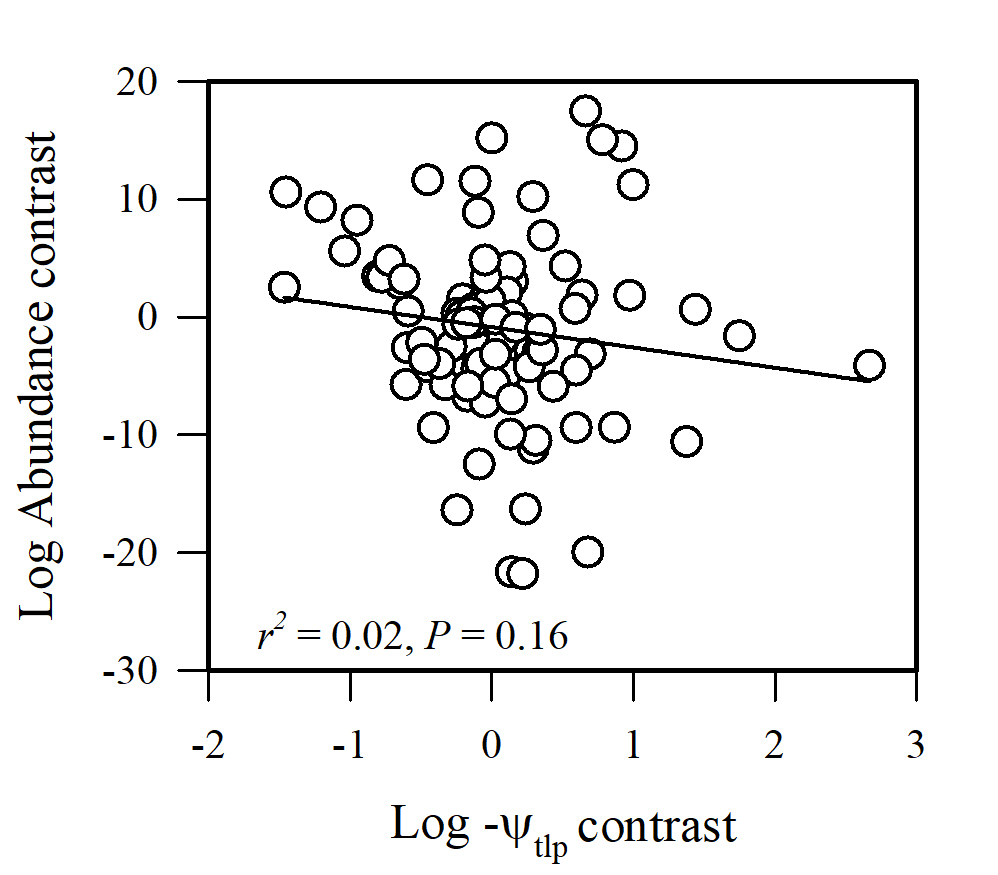
**

**Fig. S4**

**
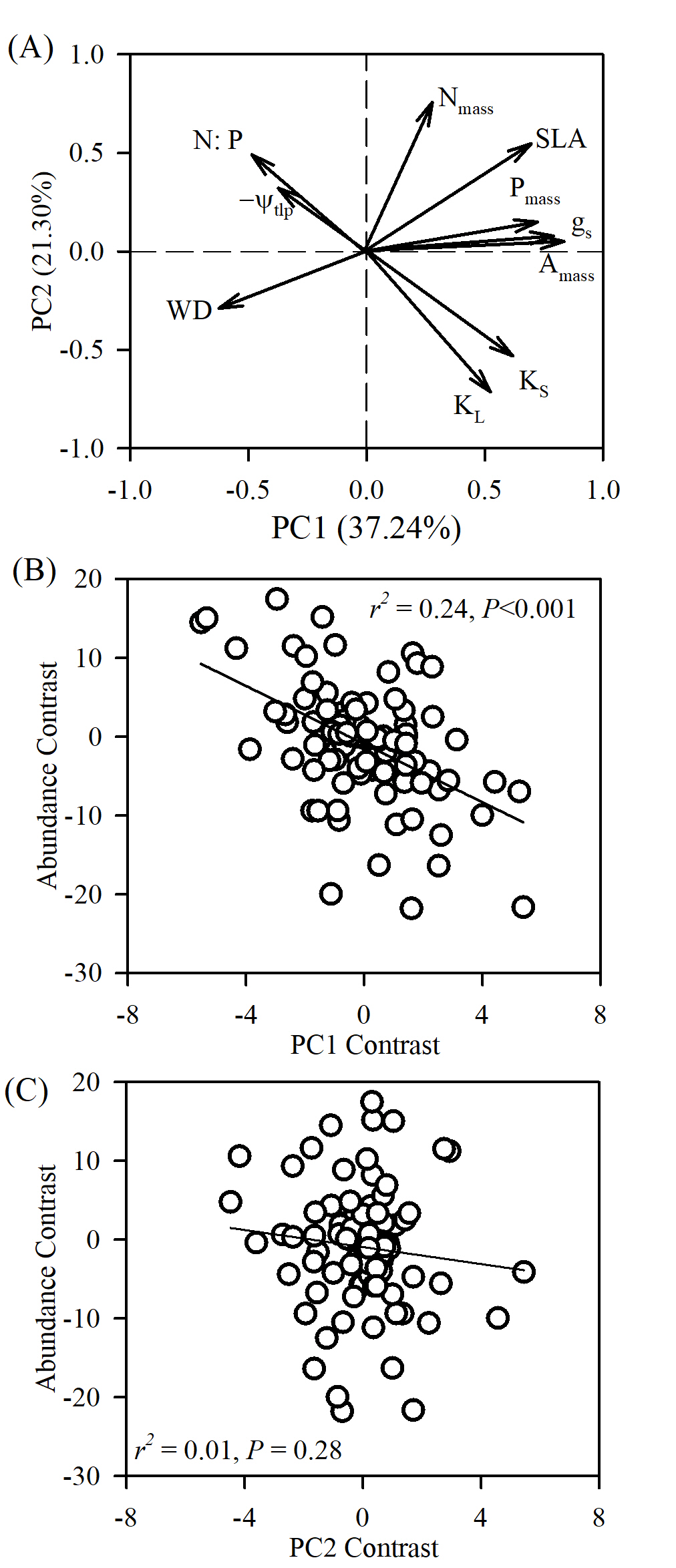
**

**Fig. S5**


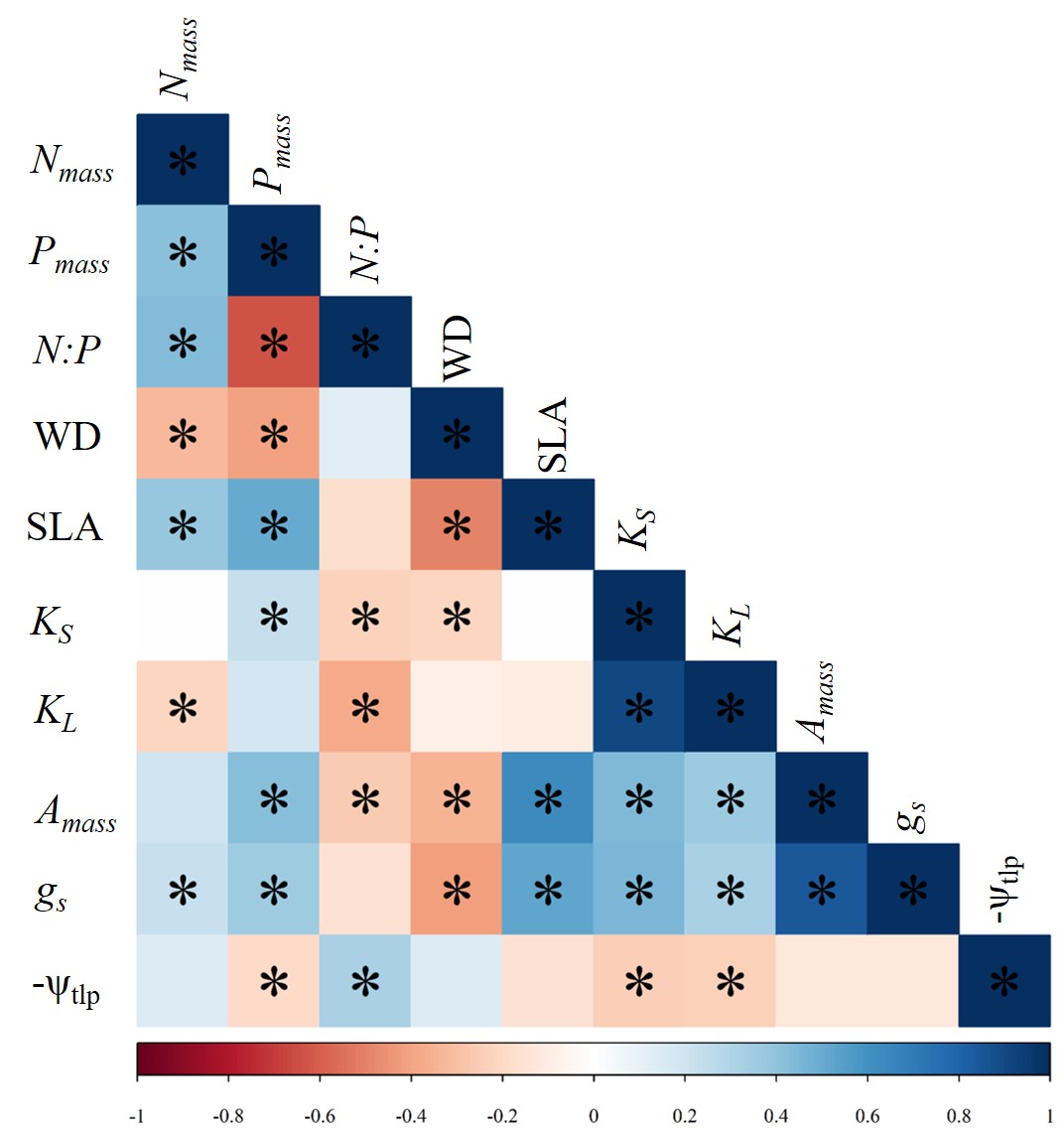


**Fig. S6**

**Table S1**. Results of the multiple regression for plant functional traits and species abundance based on PICs.

| Models | *N* | *B* | SE *B* | *t* | *F* | *R*^2^ | *P* |
| --- | --- | --- | --- | --- | --- | --- | --- |
| *g_s_* + *P_mass_* +K_S_+K_L_+ψ_tlp_ | 101 |  |  |  | 14.69 | 0.44 | <0.0001 |
| *g_s_* |  | -2 | 0.39 | -5.2 |  |  | <0.0001 |
| *P_mass_* |  | -2.95 | 0.80 | -3.68 |  |  | <0.001 |
| *K_S_* |  | 2.72 | 0.71 | 3.85 |  |  | <0.001 |
| *K_L_* |  | -2.36 | 0.56 | -4.2 |  |  | <0.0001 |
| −ψ_tlp_ |  | -3.1 | 0.98 | -3.2 |  |  | 0.002 |

*Notes*: Stomatal conductance per unit mass (*g_s_*); leaf phosphorus content (*P_mass_*); sapwood-specific hydraulic conductivity (*K_S_*) and leaf-specific hydraulic conductivity (*K_L_*) and turgor loss point (-ψ_tlp_).
